# Phosphorylation of Mitogen- and Stress-Activated Protein Kinase-1 in Astrocytic Inflammation: A Possible Role in Inhibiting Production of Inflammatory Cytokines

**DOI:** 10.1371/journal.pone.0081747

**Published:** 2013-12-11

**Authors:** Peipei Gong, Xide Xu, Jinlong Shi, Lanchun Ni, Qingfeng Huang, Liang Xia, Dekang Nie, Xiaojian Lu, Jian Chen, Wei Shi

**Affiliations:** Department of Neurosurgery, Comprehensive Surgical Laboratory, Affiliated Hospital of Nantong University, Nantong University, Nantong, Jiangsu Province, P.R.China; Albany Medical College, United States of America

## Abstract

**Purpose:**

It is generally accepted that inflammation has a role in the progression of many central nervous system (CNS) diseases, although the mechanisms through which this occurs remain unclear. Among mitogen-activated protein kinase (MAPK) targets, mitogen- and stress-activated protein kinase (MSK1) has been thought to be involved in the pathology of inflammatory gene expression. In this study, the roles of MSK1 activation in neuroinflammation were investigated.

**Methods:**

The bacterial lipopolysaccharide (LPS)-induced brain injury model was performed on Sprague-Dawley rats. The dynamic expression changes and the cellular location of p-MSK1 in the brain cortex were detected by Western blot and immunofluorescence staining. The synthesis of inflammatory cytokines in astrocytes was detected by enzyme-linked immunosorbent assay (ELISA).

**Results:**

Phosphorylated MSK1 (p-MSK1 Thr-581) was induced significantly after intracerebral injection of LPS into the lateral ventricles of the rat brain. Specific upregulation of p-MSK1 in astrocytes was also observed in inflamed cerebral cortex. At 1 day after LPS stimulation, iNOS, TNFα expression, and the astrocyte marker glial fibrillary acidic protein (GFAP) were increased significantly. Also, *in vitro* studies indicated that the upregulation of p-MSK1 (Thr-581) may be involved in the subsequent astrocyte inflammatory process, following LPS challenge. Using an enzyme-linked immunosorbent assay (ELISA), it was confirmed that treatment with LPS in primary astrocytes stimulated the synthesis of inflammatory cytokines, through MAPKs signaling pathways. In cultured primary astrocytes, both knock-down of total MSK1 by small interfering RNAs (siRNA) or specific mutation of Thr-581 resulted in higher production of certain cytokines, such as TNFα and IL-6.

**Conclusions:**

Collectively, these results suggest that MSK1 phosphorylation is associated with the regulation of LPS-induced brain injury and possibly acts as a negative regulator of inflammation.

## Introduction

Emerging evidence indicates that the inflammatory response in the brain represents a potential pathogenic factor in many central nervous system (CNS) diseases, including chronic neurodegenerative diseases, such as Alzheimer’s disease (AD), Parkinson’s disease (PD), ischemic brain injury (IBI), and even traumatic brain injury (TBI) [1]. It is widely believed that the accumulation of inflammatory cells and the production of pro-inflammatory cytokines contribute to a number of different pathological states within the CNS, including injury, ischemia, infection, and neurodegenerative diseases [2]. With further research, accumulating epidemiological evidence implicates traumatic brain injury (TBI) as a potential risk factor for AD or PD [3], [4]. It is clear from such evidence that the inflammatory response plays an important role in CNS disease.

A hallmark of neuroinflammation is the activation of glial cells, including astrocytes and microglial cells [5]. Despite obvious differences in morphology and functional properties, these cells are regarded as immune active cells and in some instances, they share common innate immune responses. For example, both astrocytes and microglial cells have been shown to respond to pro-inflammatory cytokines and lipopolysaccharides (LPS) in the induction of iNOS and other inflammatory factors [6]–. In the past few years, it was thought that microglia, as the primary immune cell in the CNS, played a key role in inflammatory processes in the brain [5]. However, increasing evidence points to the potential of reactive astrogliosis to play important roles in the pathological process of neuroinflammation [8], [9].

Astrocytes are the major glial cell population within the adult CNS. They have been proposed to exert a wide range of essential complex functions, including guidance of the development and migration of neurons during brain development, production of growth factors, maintenance of the integrity of the blood–brain barrier, and participating in the immune and repair responses to disease and brain injury [10]–[12]. Traumatic injury to the adult CNS results in a rapid inflammatory response by the resident astrocytes, characterized primarily by hypertrophy, proliferation, and increased glial fibrillary acidic protein (GFAP) expression, resulting in the release of inflammatory and cytotoxic substances [13]–[15]. Thus, a balance between pro- and anti-inflammatory signaling arising from active astrogliosis within the affected brain area will eventually determine the outcome of the CNS inflammatory process.

Mitogen-activated protein kinases (MAPKs) can be activated by a wide variety of different stimuli, and, generally, their functions are mediated through phosphorylation of several substrates, including extracellular signal-regulated kinase (ERK) and p38 MAPK [16]. Once activated, the MAPKs phosphorylate their respective substrates, including several nuclear and cytoplasmic targets, regulating diverse cellular responses, including cell proliferation, differentiation, survival, apoptosis, and the inflammatory response [17]–[19]. Among the MAPK targets, mitogen- and stress-activated protein kinase 1 (MSK1) is activated downstream of p38 and ERK1/2, indicating that both mitogens and stress stimuli lead to the activation of MSK1 [20]. Several in vitro studies have demonstrated that MSK1 can induce the phosphorylation of CREB and activation of NF-κB, both of which are key regulators of the transcription of a variety of genes involved in immune and inflammatory responses [21], [22]. The role of MSK1 in the inflammatory process has been discussed widely. For example, in macrophages, it was recently shown that MSK1 is involved in negative feedback pathways that are crucial in preventing uncontrolled inflammation [23]. It is also known that glucocorticoid (GC) hormones are able to provoke a subcellular redistribution of MSK1 to the cytoplasm, as a result of an anti-inflammatory effect [24]. In response to LPS, expression of the anti-inflammatory cytokine interleukin (IL)-10 was upregulated in wild-type bone marrow-derived macrophages, but not in MSK1-knockout cells [23]. In this case, MSK1 targets both pro- and anti-inflammatory genes during the process of inflammation. However, how this kinase operates on a tight equilibrium of positive and negative feedback mechanisms in inflammation remains unknown.

The activation mechanism of MSK1 has not been extensively studied. In cells, MSK1 is activated via a complex series of phosphorylation and auto-phosphorylation reactions, downstream of ERK or p38 MAPK, depending on the stimulus used [25], [26]. In addition to being phosphorylated on Thr-581 and Ser-360 by ERK or p38, MSK1 can autophosphorylate on at least six other sites. Of them, Thr-581 and Ser-360 were reported to be essential for the catalytic activity of MSK1 and thus for the phosphorylation of MSK1 substrates [25]. Despite significant correlative evidence of a role for total MSK1 expression in regulating inflammation, direct genetic evidence supporting the role of the respective phosphorylation site of MSK1 in CNS inflammation and injury has not yet been reported. Indeed, little is known about whether activating MSK1 phosphorylation sites is associated with inflammatory cytokine release, regulating the astrocyte inflammatory response.

This study may provide insights into the regulation of MSK1 production and phosphorylation site activation following LPS injection in the brain, modeling conditions that may occur in vivo in pathological situations, such as CNS inflammation and injury.

## Materials and Methods

### Experimental Animals and Treatment

All surgical interventions and postoperative animal care were carried out in accordance with the Guide for the Care and Use of Laboratory Animals (National Research Council, 1996, USA) and were approved by the Chinese National Committee to the Use of Experimental Animals for Medical Purposes, Jiangsu Branch. All efforts were made to minimize the number of animals used and their suffering. Male Sprague-Dawley rats (180–220 g; Experimental Animal Center, Nantong University, China) were caged in groups of three with food and water available *ad libitum*. The animals were kept in a temperature-controlled environment (21°C) on a 12/12-h light/dark cycle. Surgery was performed as described elsewhere [27]. Briefly, the rats were deeply anesthetized with chloral hydrate (10% solution) and surgery was performed under aseptic conditions. The right lateral ventricle was reached stereotaxically (David Kopf Instruments, Tujunga, CA) using the Paxinos & Watson atlas (1986) [45]. With the incisor bar placed at 3.3 mm below the interaural line (horizontal zero), the coordinates from bregma for the guide cannula were 1.0 mm anteroposterior, 1.5 mm lateral, and 3.8 mm dorsoventral. A 22-gauge stainless steel guide cannula was implanted close to the right lateral ventricle and secured with screws and cranioplastic cement (cranioplastic powder, Plastic One, Roanoke, VA; repair material, Dentsply International, York, PA). Lipopolysaccharides which purchased from Sigma Chemicals are purified by phenol extraction. The serotype of LPS used in this study is Escherichia coli 055:B5. The article number of LPS is L2880. And 0.5 µl of LPS was injected at each rat. The rats were allowed to recover from surgery. Later, the animals were anesthetized to harvest the cerebral cortex at 1, 3, 6, 9, or 12 h, or 1, 3, or 5 days after injury.

### Cell Culture and Cell Treatment

Primary astrocytes were prepared from the cerebral cortex of 1–2-day-old neonatal Sprague-Dawley rat pups as described previously. Briefly, the rat pups were decapitated and the brains were removed rapidly. The cerebral cortices were trypsinized, dissociated by gentle trituration, and plated at a density of 5×10^7^ cells per 75 cm^2^ flask in Dulbecco’s modified Eagle’s medium (DMEM) with nutrient mixture F12 (1∶1 *v*/*v*, Gibco, Grand Island, NY, USA) containing 10% heat-inactivated fetal bovine serum (FBS). Cells were maintained in complete culture medium for 7–8 days. Prior to experimental treatments, cultures of astrocytes were passaged twice. The culture medium was switched to serum-free DMEM/F12 culture medium and experiments were initiated 24 h later. To investigate the effect of lipopolysaccharide (LPS) on the phosphorylation of MSK1, cells were allowed to reach 80% confluence. At the time of treatment, LPS carried by saline was added to the culture medium. The infusion rate was based on the concentration of LPS used in each study. The reagents were administered directly into growth media for the indicated incubation times. Non-treated cells were included as controls in all experiments. In this culture, more than 98% of the cells were identified as type I astrocytes by positive activity for GFAP and by their flattened, polygonal appearance.

### siRNAs and Transfection

Primer pairs for the rat MSK1 (XM_213209.4) siRNA expression vector were made to target the sequence CATCAGCCACGAGACATCA. The rat MSK1siRNA expression vector was successfully constructed. The non-silencing control siRNA was an irrelevant siRNA with random nucleotides of no known specificity. Transfection of rat primary astrocytes with duplex synthetic siRNA was performed using lifectamine 2000 reagents (Invitrogen) according to the manufacturer’s instructions. For transient transfection, the MSK1 siRNA vector and the non-specific vector were transfected using Lipofectamine 2000 (Invitrogen) and plus reagent in OptiMEM (Invitrogen), as suggested by the manufacturer. Transfected cells were used for the subsequent experiments 48 h after transfection. The transfenction efficiency was measured by western blot analysis. The concentration of siRNA used was 20 uM.

### Plasmid Construction and Site-directed Mutagenesis

The full-length human MSK1 complementary DNA (cDNA) was amplified by PCR using two oligonucleotides and cloned into the *Bgl*II-*Eco*RI sites of plasmid pEGFP-N2 (Clontech, Palo Alto, CA), generating the pEGFP-MSK1wt vector. Human cDNAs encoding a non-phosphorylatable mutant with a threonine-to-alanine substitution at position 581 (p-MSK1 (T581A)) was introduced into the pEGFP-MSK1wt vector using the overlap extension PCR technique. The primers for replacing Thr581 with the Ala (T581A) mutant were 5′-GATAATCAGCCCCTGAAGGCTCCATGCTTCACCCTTC-3′ and 5′-GAAGGGTGAAGCATGCCTTCAGGGGCTGATTATC-3′. For transient expression experiments, MSK1 digests from the pEGFP-MSK1wt and pEGFP-MSK1 (T581A) plasmids were inserted into the Xho*I*/Eco*RI* sites of the pcDNA3.1/myc-His expression vector (Invitrogen, Carlsbad, CA). All MSK1 cDNA constructs were sequenced fully to ensure the absence of cloning artifacts.

### Western Blotting Analysis

Western blotting was conducted with tissue from normal cerebral cortex or from inflamed cerebral cortex at 1, 3, 6, 9, or 12 h, or 1, 3, or 5 d after injection of LPS (4 or 5 rats per group). To obtain samples for Western blotting, the normal or inflamed cerebral cortex segments were excised and snap-frozen at −70°C until utilized. The samples were then homogenized in lysis buffer (1% NP-40, 50 mmol/L Tris, pH 7.5, 5 mmol/L EDTA, 1% SDS, 1% sodium deoxycholate, 1% Triton X-100, 1 mmol/L PMSF, 10 µg/mL aprotinin, and 1 µg/mL leupeptin) and clarified by centrifugation (20 min in a microcentrifuge, 4°C). After appropriate stimulation, cells were washed twice with ice-cold D-Hanks and extracted in lysis buffer for 45 min on ice. After determination of the protein concentration using the Bradford assay (Bio-Rad), the resulting supernatant was subjected to SDS-polyacrylamide gel electrophoresis (PAGE). The separated proteins were transferred to a polyvinylidine difluoride membrane (Millipore) by a transfer apparatus (350 mA for 2.5 h). The membrane was then blocked with 5% nonfat milk and incubated with primary antibodies against MSK1 (rabbit, 1∶1000; Novus Biologicals), Phospho-MSK1 (Thr581) (rabbit, 1∶1000; Cell-Signalling), Phospho-MSK1 (Ser360) (rabbit, 1∶5000; abcam), iNOS, TNFα, GFAP (mouse, 1∶1000; Sigma), or GAPDH (mouse, 1∶1000; Sigma). After incubating with an anti-rabbit or anti-mouse horseradish peroxidase-conjugated secondary antibody, proteins were visualized using an enhanced chemiluminescence system (ECL, Pierce Company, USA).

### Sections and Immunofluorescent Staining

After defined survival times, rats were terminally anesthetized and perfused through the ascending aorta with 500 mL of 0.9% saline, followed by 4% paraformaldehyde. After that, the brains were removed and postfixed in the same fixative for 3 h, which was then replaced with 20% sucrose for 2–3 days, followed by 30% sucrose for 2–3 days. After treatment with sucrose solutions, the tissues were embedded in OTC compound. Then, 10 µm frozen cross-sections were obtained by freezing microtome. All sections were examined at coronary position. Sections were stored at −20°C until utilized. All sections were first blocked with 10% normal serum blocking solution of the same species as the secondary antibody, containing 3% (w/v) bovine serum albumin (BSA), 0.1% Triton X-100, and 0.05% Tween-20 for 2 h at room temperature to reduce non-specific staining. Then, the sections were incubated with mouse monoclonal primary antibodies for anti-NeuN (a marker for neurons, 1∶600 or 1∶500; Chemicon), anti-GFAP (a marker for astrocytes, 1∶200; Sigma) and anti-TNFα and affinity-purified goat polyclonal primary antibodies for anti-MSK1 (1∶100; Santa Cruz) or anti-pMSK1 (Thr581, 1∶100; Cell-Signalling). Briefly, sections were incubated with both primary antibodies overnight at 4°C, followed by a mixture of FITC- and TRITC-conjugated secondary antibodies for 2 h at 4°C. Stained sections were examined with a Leica fluorescence microscope (Germany).

### Coimmunoprecipitation

Primary astrocytes were seeded at 1×10^6^ per dish and grown until about 80% confluence. After 48 h of starvation, cells were treated as indicated in the figure legends. Cells were promptly homogenized in a homogenization buffer (50 mmol/L Tris-HCl (pH 7.5), 150 mmol/L NaCl, 1% Triton X-100, 1% NP-40, 10% SDS, 0.5% sodium deoxycholate, 5 mmol/L EDTA, 10 µg/ml leupeptin, 10 µg/ml aprotinin, and 1 mmol/L PMSF), then centrifuged at 12,000 rpm for 20 min at 4°C to collect the supernatant. Immunoprecipitation were carried out by incubating 0.5 mg of total cell lysates with primary antibody (anti-MSK1) at 4°C overnight. Protein G-Sepharose (Sigma, 1∶1 slush in PBS) was then added for 2 h at 4°C wheel rocking. The precipitates were washed four times with ice-cold PBS, resuspended in SDS sample buffer, and resolved by SDS-PAGE followed by immunoblot analysis.

### Inhibitors and Cytokine Measurements

Specific inhibitors of p38, ERK and MSK1 were purchased from Sigma Chemicals. Astrocytes were pretreated with PD98059 (50 µM), SB203580 (20 µM), or H89 (10 µM) for 1 h and stimulated with 1 µg/mL LPS for 12 h. The best concentrations of the individual inhibitors were obtained from the manufacturer’s specifications. The carrying solution of PD98059 or SB203580 was DMSO. And the carrying solution of H89 was ddH_2_O. Concentrations of IL-6, IL-1β, and TNFα in the culture supernatant were determined using enzyme-linked immunosorbent assay (ELISA) kits (Biosource Europe, SA). The detection limits of the assays were determined to be 15 pg/mL. The assays were performed according to the manufacturer’s protocols.

### Quantitative Analysis

Cells double-labeled for p-MSK1 (Thr581) and the other phenotypic markers used in the experiment were quantified. Sections were double-labeled for p-MSK1 (Thr581) with GFAP and NeuN. To identify the proportion of each phenotype-specific marker, in positive cells expressing p-MSK1 (Thr581), a minimum of 200 phenotype-specific marker-positive cells were counted in the cerebral cortex. Then, double-labeled cells for p-MSK1 (Thr581) and the phenotype-specific marker were recorded. Cell quantification in the cortex was performed in an unbiased manner according to the principles described by Konigsmark (1970). To avoid counting the same cell in more than one section, we counted every fifth section (50 µm apart). Investigators were blinded to treatment group to avoid subjective assume during quantification.

### Statistical Analyses

The statistical graphs of western blotting results were obtained by comparing the relative optical density of the target proteins and reference protein. And we calculated the relative optical density by software called Image J. Quantitative data are presented as mean ± SEM. Cell count data were analysed with overall distribution of chi-square test. All remaining data were analysed using student’s t -test or one-way ANOVA, where appropriate. All statistical tests were performed using the GraphPad Prism Program, Version 3.02 for Windows (GraphPad Software, Inc., San Diego, CA) and SPSS 11.0 for Windows (SPSS, Inc., Chicago, Illinois). P values<0.05 were deemed to indicate statistical significance. Each experiment consisted of at least three replicates per condition.

## Results

### 1. Expression Profile of MSK1 and p-MSK1 following Intracerebroventricular Injection of LPS

**Figure 1:**
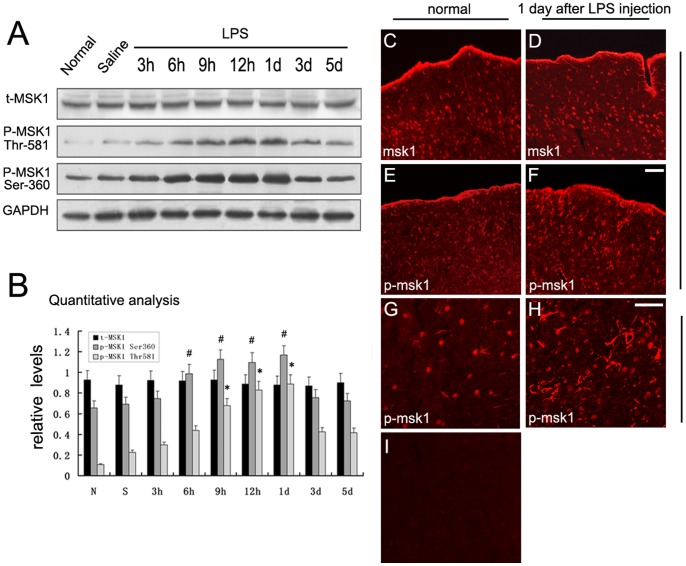
Expression profile of MSK1 and p-MSK1 (Thr-581 and Ser-360) following LPS intracerebral injection. **A**. Protein levels of t-MSK1, p-MSK1 Thr-581, p-MSK1 Ser-360 were detected before (control) and after injury. GAPDH was also detected by Western blotting. **B**. Quantification graphs (relative optical density) of the intensity of staining of p-MSK1 (Thr-581) and total MSK1 to GAPDH at each time point. GAPDH was used to confirm that equal amounts of protein were run on the gel. **C–H**. Immunofluorescence staining of MSK1 and p-MSk1 (Thr581) was performed to assess the staining changes for MSK1 and p-MSK1 immunoreactivity in the cortex at day 1 after LPS-injection. **I**. Negative control. * and ^#^ indicate significant differences at P<0.05, compared with normal brain cortex. Scale bars: 40 µm (C–F), 20 µm (G–J).

To investigate temporal patterns of MSK1 and p-MSK1 (Thr-581 and Ser-360) expression after CNS inflammation, Western blotting was performed. The results revealed that p-MSK1 (Thr-581 and Ser-360) expression was increased significantly, compared with controls. In particular, Thr-581-phosphorylated MSK1 was low in normal cortex, increased progressively from 6 h after LPS injection, peaked at 12 h to 1 day, and then declined gradually. Serine-360-phosphorylated MSK1 (p-MSK1S360) was detectable in normal brain. After LPS administration, p-MSK1S360 also showed a similar expression pattern to p-MSK1T581 in a time-dependent manner. Next, we assessed the expression of MSK1 and p-MSK1 (Thr-581 and Ser-360) in brain cortex after saline injection, and we found their expression was unchanged at any time tested. On the other hand, there was no obvious difference in MSK1 expression among the time points tested in this study (Fig. 1A, B). This may suggest that MSK1 can exert biological function(s) after being phosphorylated by LPS. To further confirm the results of the Western blot analysis, we also performed single immunofluorescence staining to determine staining changes in MSK1 and p-MSK1 (Thr-581) immunoreactivity in the cortex at day 1 after LPS-injection. In naïve controls, p-MSK1T581 immunoreactivity was observed at low levels in the cortex (Fig. 1E). At 1 day after LPS injection, p-MSK1 (Thr-581) staining was obviously increased in the brain cortex (Fig. 1F). Furthermore, higher magnification of the cortex revealed clearly that p-MSK1 (Thr-581) expression at day 1 after LPS-injection higher in cells with a star-shaped, astrocytic morphology (Fig. 1G, H). Also, MSK1 immunoreactivity showed no change (Fig. 1C, D).

### 2. Immunolocalization of MSK1 and p-MSK1 (Thr-581) with different Cellular Markers in the Cerebral Cortex

**Figure 2:**
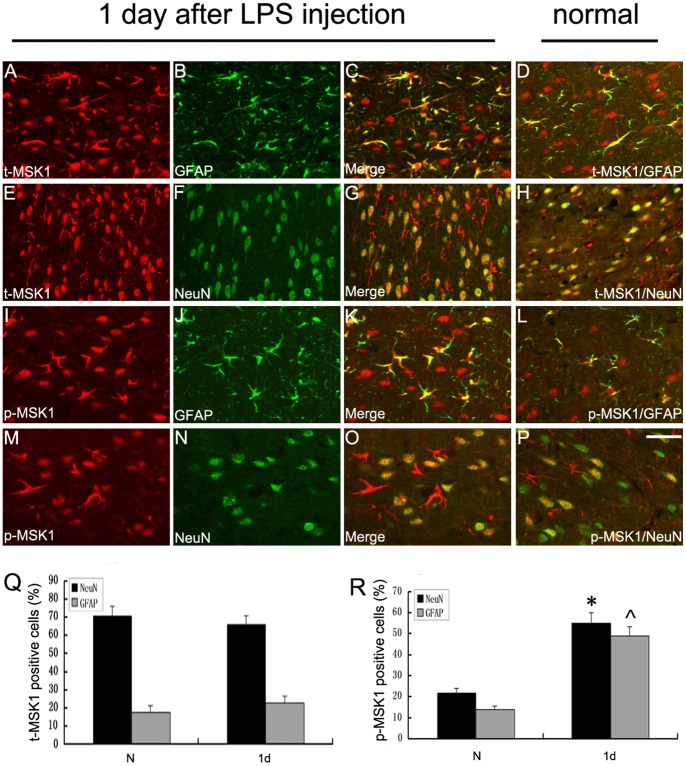
Immunolocalization of MSK1 and p-MSK1 (Thr-581) with different cellular markers in cerebral cortex by double immunofluorescence staining. In the adult rat brain cortex, within 5(red, **A** and **E**) and p-MSK1 (Thr581) (red, **I** and **M**) and different cell markers (green, **B, F, J, N**), such as a neuronal marker (NeuN) and an astrocyte marker (GFAP). The yellow color in the merged images represents colocalization of MSK1 or p-MSK1 (Thr581) with different phenotype-specific markers (**C, G, K, O**). Colocalization of MSK1 and p-MSK1 (Thr581) with different phenotype-specific markers in the normal group are shown in the brain cortex (**D, H, L, P**). Quantitative analysis of different phenotype-specific marker-positive cells expressing MSK1 (**Q**) and p-MSK1 (**R**) (%) in the unit area (mm^2^) in the normal group and 1 day after injury. *indicates significant difference at P<0.05, compared with the normal group. Error bars indicate SEM. Scale bars: 20 µm (**A–P**).

To further confirm the cellular localization of p-MSK1T581 and t-MSK1 (total MSK1), double-labeling immunofluorescent staining was performed with cell-specific markers (Fig. 2). We found that t-MSK1 was widely expressed, mainly in neurons and with a relative low level in astrocytes in normal brain (Fig. 2D, H). In the injured brain cortex, t-MSK1 expression was unchanged in neurons or in activated astrocytes (Fig. 2A–H). However, the results also showed a low level of expression of p-MSK1 (Thr-581) in neurons and/or unactivated astrocytes (Fig. 2L, P). In the group 1 day after LPS administration, a significant increase in p-MSK1T581 immunoreactivity was observed in astrocytes compared with the normal group (Fig. 2I–L) As shown in Figure 2, there was also an increase in p-MSK1T581 immunoreactivity in neurons after LPS-injection (Fig. 2M–P). By quantitative analysis, p-MSK1T581 expression was increased significantly in astrocytes and neurons at 1 day after LPS injection (Fig. 2R). No obvious alteration in t-MSK1 expression was observed, regardless of the injection (Fig. 2Q).

### 3. Involvement of p-MSK1Thr-581 in Astrocytic Inflammation after LPS Injection

**Figure 3:**
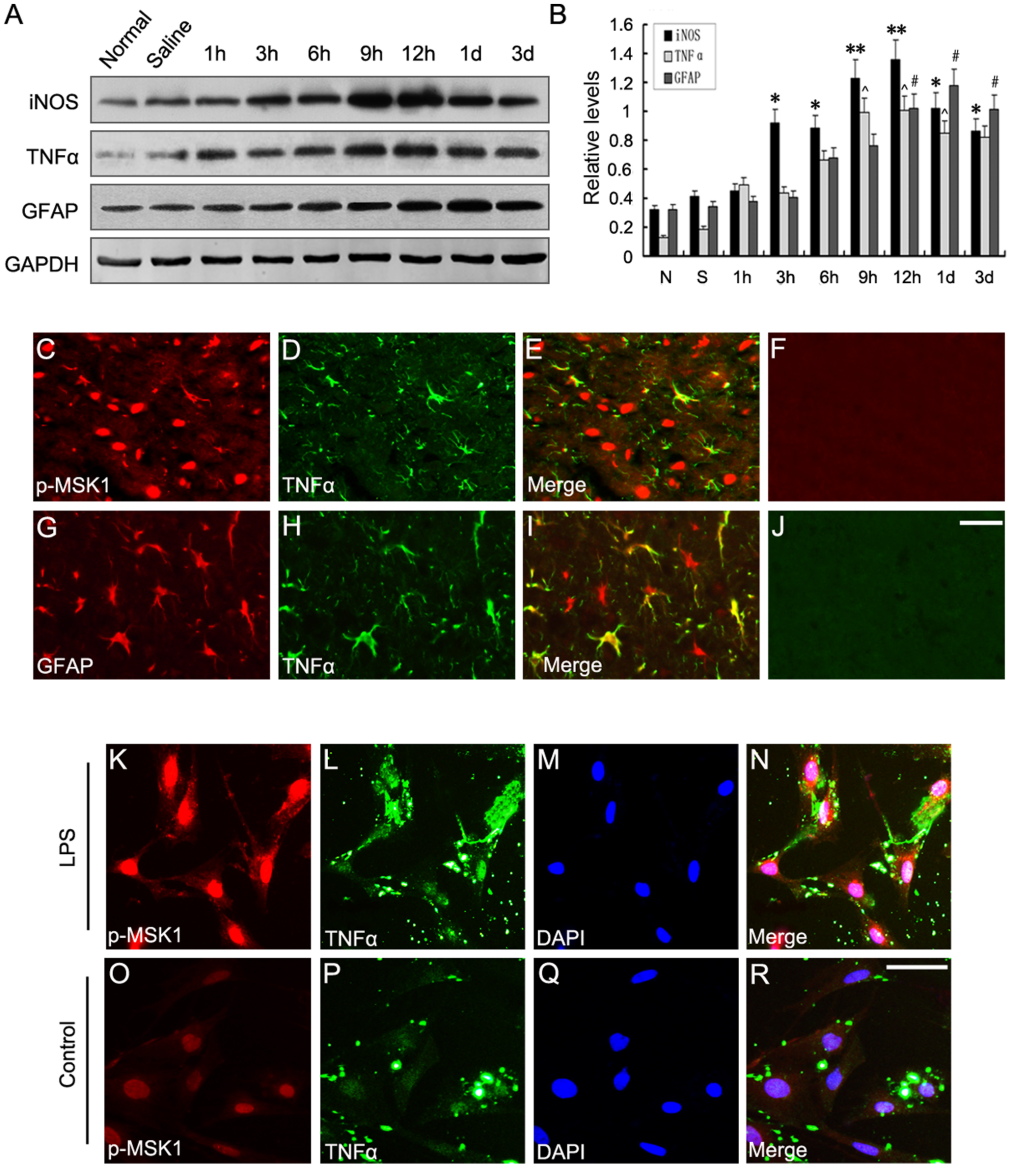
Expression of pro-inflammatory mediators by active astrocytes after LPS treatment. **A**. Western blot analysis showed the time course of TNFα, iNOS, and GFAP protein expression in LPS-treated rats. **B**. Quantification graphs (relative optical density) of the intensity of staining of iNOS, TNFα, and GFAP to GAPDH at each time point. GAPDH was used to confirm that equal amounts of protein were run on the gel. The data are means ± SEM (*n* = 3, **, P<0.01; *, P<0.05; ^#^, P<0.01; ^∧^, P<0.01, significantly different from the normal group). **C–J**. Double immunofluorescence staining for TNFα, GFAP, and p-MSK1 (Thr581) in brain cortex 1 day after LPS injection. The yellow color in the merged images indicates colocalization of TNFα with GFAP and p-MSK1. **K–R**. Immunofluorescent staining for TNFα and p-MSK1 (Thr581) in primary astrocyte cultures treated with (I–L) or without (M–P) LPS. **F** and **J**: Negative control. Scale bars: 20 µm (C–H).

Intracerebroventricular injection of LPS is an established model of neuroinflammatory processes associated with reactive astrogliosis [28]. After injection of LPS, markers of glial reactivity were accentuated, including increased activation of astrocytes (GFAP+), upregulated levels of proinflammatory mediators (TNFα/iNOS) in the lesion area in a time-dependent manner, as demonstrated by the Western blot analysis (Fig. 3A, B). Meanwhile, to identify the inflammatory cell types present after intracerebral injection of LPS, double immunofluorescence staining was performed with p-MSK1Thr-581, GFAP, and TNFα on injured brain sections. It is already known that increased expression of p-MSK1 after LPS administration is primarily in astrocytes and neurons. The results here suggest an increased number of TNFα-positive astrocytes (Fig. 3G–I). Additionally, astrocytic inflammation, evaluated by TNFα, appeared in many p-MSK1Thr-581-expressing cells at day 1 after LPS injection (Fig. 3C–E). Furthermore, cell immunofluorescence staining was performed to investigate the sublocalization of p-MSK1Thr-581 and proinflammatory mediators. We found that, regardless of stimulation, there was no obvious change of the sublocation of p-MSK1 (Thr-581). LPS can also lead to enhanced intensity of p-MSK1 (Thr-581) and TNFα fluorescence (Fig. 3K–R). Based on these results, we concluded that, p-MSK1 (Thr-581) may be associated with the process of TNFα-dependent astrocytic inflammation, induced by LPS.

### 4. Expression of p-MSK1 in Activated Astrocytes after LPS Exposure *in vitro*

**Figure 4:**
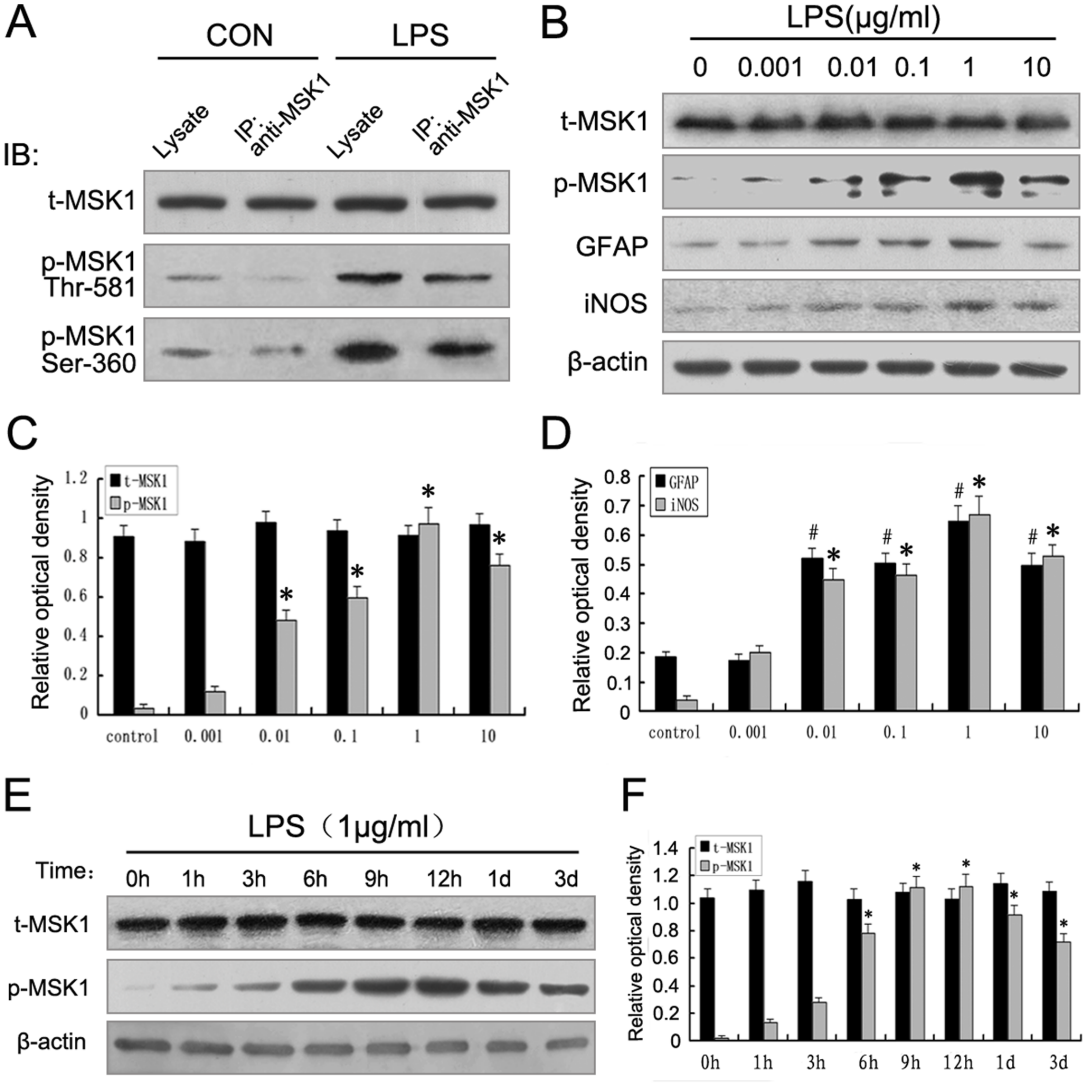
Phosphorylation of MSK1 induced by LPS in cultured primary astrocytes. **A**. Endogenous MSK1 was immunoprecipitated (IP) from astrocytes and treated with LPS. The concentration of LPS was 1 µg/ml and the stimulation time was 9 h. Immunoprecipitates were run on SDS gels and blotted for total MSK1, phospho-Thr-581, and phospho-Ser-360. **B**. Western blot analysis showed that LPS induced astrocytes to express p-MSK1 (Thr581), GFAP, iNOS, and total MSK1 in a dose-dependent manner. **C**. The bar chart shows the ratio of total MSK1 and p-MSK1 Thr-581 to β-actin. **D**. The bar chart shows the ratio of GFAP and iNOS to β-actin. **E**. Western blot analysis showed that LPS induced astrocytes to express p-MSK1 (Thr-581) in a time-dependent manner. **F**. The bar chart shows the ratio of total MSK1 and p-MSK1 to β-actin.

We have found that p-MSK1 (Thr-581 and Ser-360), activated by upstream kinases, might be involved in the process of astrocytic inflammation via the phosphorylation cascade after LPS injection. To further prove that activation of these phosphorylation sites in MSK1 is not the result of overexpression and do occur in the endogenous protein, a series of experiments was performed. We determined the patterns of p-MSK1 (Thr-581 and Ser-360) expression in cultured primary astrocytes following LPS exposure. To confirm that all these sites were true physiological sites, endogenous MSK1 was immunoprecipitated from untreated astrocytes and LPS-treated astrocytes and the immunoprecipitate was blotted using phospho-specific antibodies. The results showed that LPS was capable of stimulating the phosphorylation of endogenous MSK1 at Thr-581 and Ser-360 (Fig. 4A). Next, rat primary astrocytes were treated with various concentrations of LPS (0, 0.001, 0.01, 0.1, 1, 10 µg/mL) for 9 h. Using Western blotting, we then examined p-MSK1 Thr-581, GFAP, iNOS protein levels. iNOS levels began to increase at 0.01 µg/mL and gradually peaked at 1 µg/mL of LPS. In activated astrocytes, the expression of p-MSK1 (Thr-581) increased gradually in accordance with iNOS, and peaked at 1 µg/mL. Significant expression of GFAP was also observed at 0.1 and 1 µg/mL LPS. However, total MSK1 did not change significantly (Fig. 4B–D). Time-course experiments were performed at a concentration of 1 µg/mL LPS (0, 1, 3, 6, 9, and 12 h, and 1 and 3 days). Western blot analysis showed the p-MSK1 (Thr-581) protein level was low in the control group (0 h). In activated astrocytes, the expression of p-MSK1 (Thr-581) increased gradually. It rose at 6 h post-stimulus and peaked at 12 h, then declined at 1 day. However, total MSK1 expression was almost constant among the different time groups (Fig. 4E, F). These results suggest that upregulation of p-MSK1Thr-581, but not total MSK1 expression, correlated with astrocyte inflammatory response after LPS stimulation.

### 5. Effects of MAPKs Inhibitors and H89 on LPS-induced Downstream Kinase Phosphorylation and Cytokine (TNFα, IL-6, IL-1β) Production

**Figure 5:**
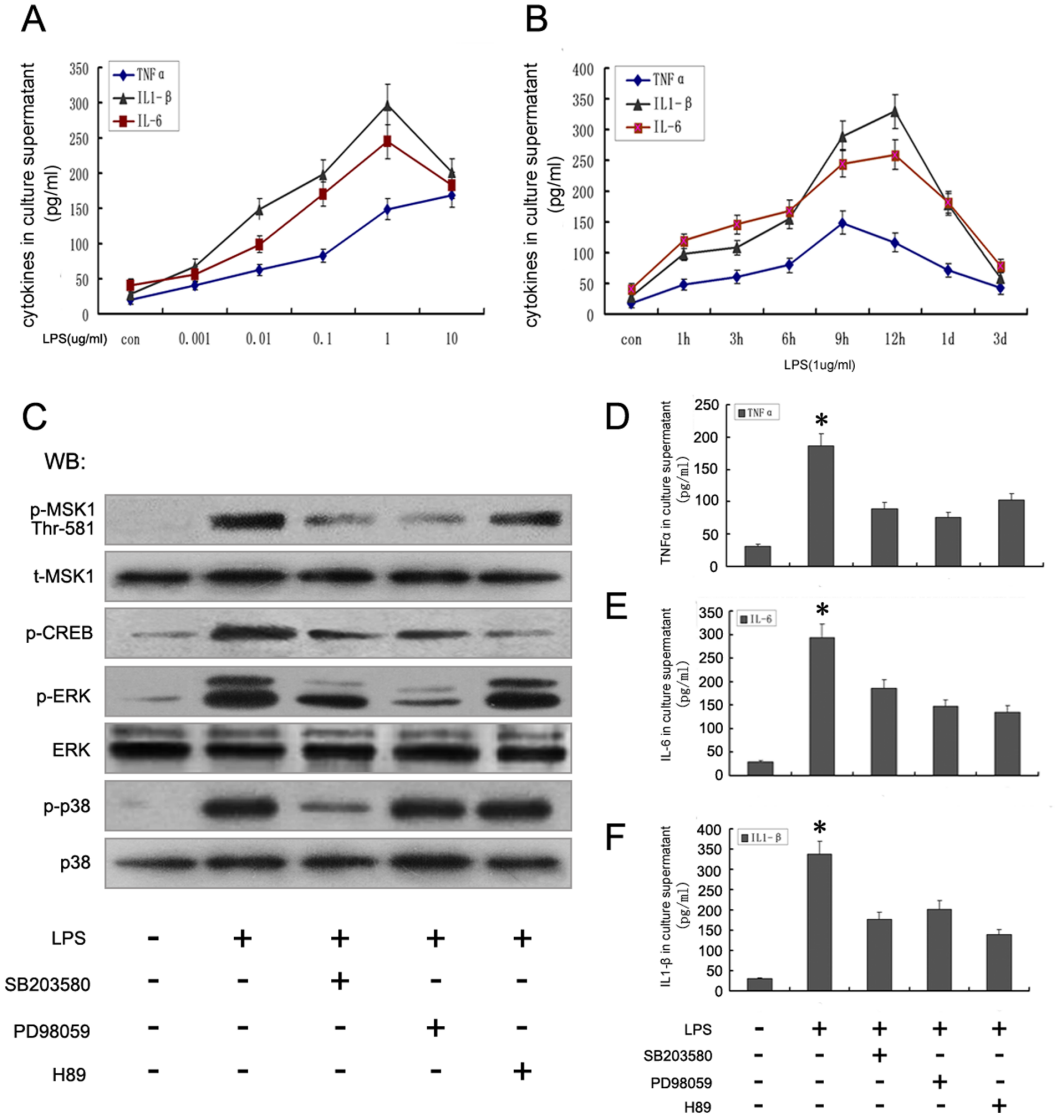
LPS promoted astrocyte inflammatory response via activating MAPKs and MSK1 pathways. **A**. ELISA analysis showed LPS induced the production of TNFα, IL-6, and IL1-β by activated astrocytes in a dose-dependent manner. **B**. ELISA analysis showed LPS induced the production of TNFα, IL-6, and IL1-β by activated astrocytes in a time-dependent manner. **C**. Astrocytes were pretreated with SB203580, PD98059, and H89 for 60 min and then stimulated with 1 µg/mL LPS for 12 h. Cell extracts were subsequently prepared and subjected to Western blotting using antibody specific for p-MSK1 (Thr581), tMSK1, p-CREB, p-ERK, ERK, p-p38, and p38. **D–F**. The TNFα, IL-6, and IL1-β contents in culture supernatants were measured by ELISA.

It has been reported that ERK and p38 MAPK are phosphorylated during various infections [29], [30]. To further understand whether activation of these two MAPK signaling pathways occurred in connection with the functions of MSK phosphorylation in astrocytes induced by LPS, their inhibitors were used. First, we detected the production of cytokines induced by LPS in astrocytes. Our observations showed that LPS induced the production of various inflammatory cytokines in astrocytes in a time- and concentration-dependent manner. Using enzyme-linked immunosorbent assay (ELISA), significant biosynthesis was observed at 12 h and the maximum response occurred after 12 h, induced with 1 µg/mL LPS (Fig. 5A, B). Then, we detected the effects of specific inhibitors, PD98059, SB203580, and H89, on the inhibition of phosphorylated ERK (pERK), phosphorylated p38 (p-p38), phosphorylated CREB (pCREB), and phosphorylated MSK Thr581 (pMSK1) activity following LPS induction in primary astrocytes. Astrocytes were pretreated with PD98059, SB203580, or H89 for 1 h and stimulated with 1 µg/mL LPS for 12 h. As shown in Figure 5, 50 µM PD98059, 20 µM SB203580, and 10 µM H89 caused significant inhibition of the expression of pERK, p-p38, and pMSK1 (Thr581), respectively. Furthermore, treatment of astrocytes with H89 significantly attenuated the ability of LPS to increase the phosphorylation of CREB (Fig. 5C). By ELISA analysis, we found that all three reagents, PD98059, SB203580, and H89, could also inhibit TNFα (Fig. 5D), IL-6 (Fig. 5E), and IL-1β (Fig. 5F) production at different degrees, induced by LPS. Thus, it seems reasonable to conclude that the MAPK (ERK, p38)-MSK pathway is involved in astrocytic inflammation induced by LPS.

### 6. Role of Phosphorylated MSK1 in Astrocytic Inflammation

**Figure 6:**
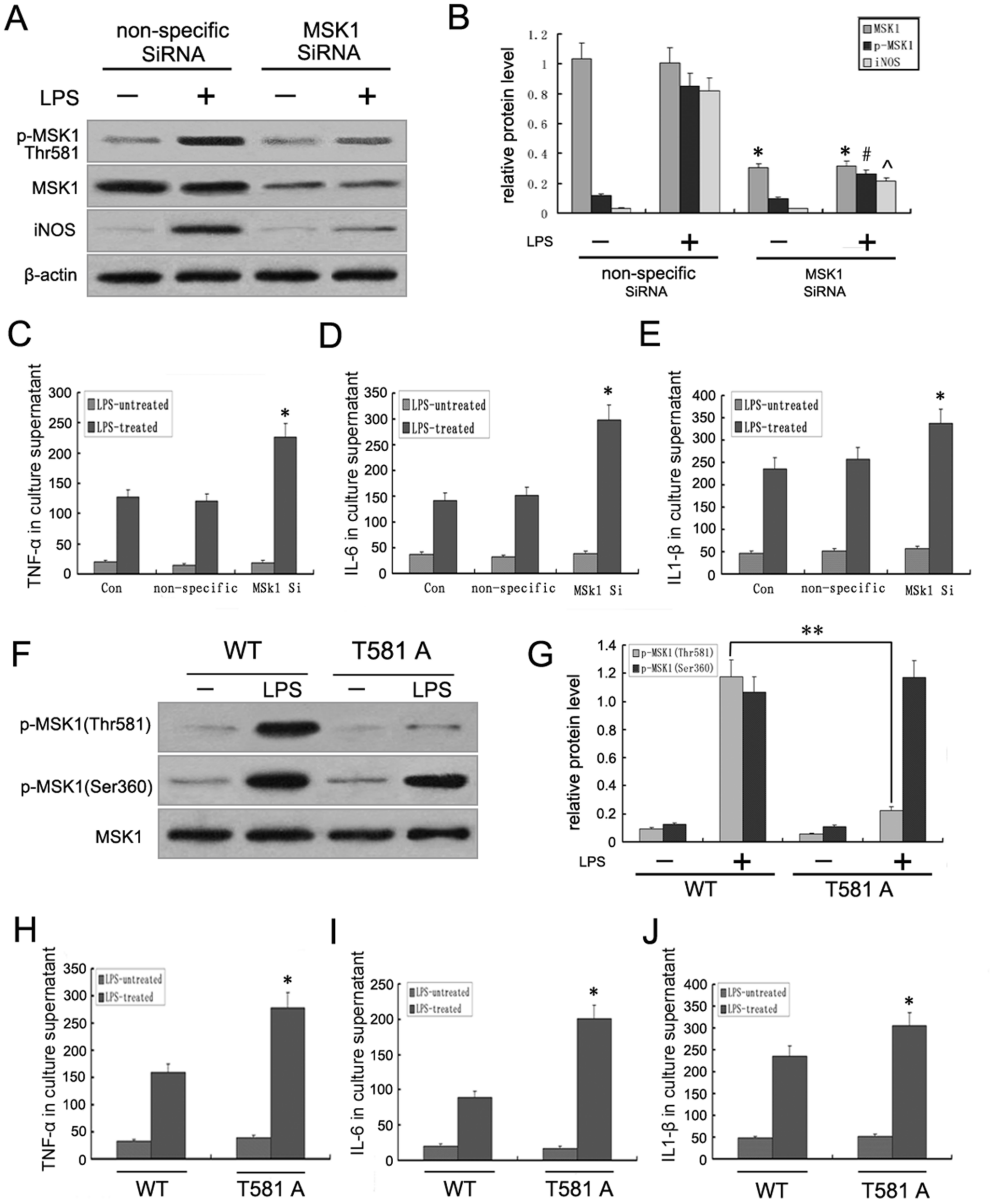
Effects of MSK1 gene silencing and Thr-581 mutation on cytokine production in LPS-treated astrocytes. **A**. Effects of siRNA for MSK1 and non-specific siRNA on LPS-induced expression of MSK1, p-MSK1 (Thr581) and iNOS were detected by Western blotting. **B**. The bar chart shows the ratio of total MSK1 and p-MSK1 to β-actin. **C–E**. ELISA showed that MSK1 gene silencing by siRNA further promoted the LPS-mediated upregulation of inflammatory cytokines. **F**. Western blot analysis showed the effect of mutation of Thr-581 to an alanine residue on LPS-induced expression of p-MSK1 Thr-581, p-MSK1 Ser-360, and total MSK1. **G**. The bar chart shows the ratio of p-MSK1 Thr-581 and p-MSK1 Ser-360 to total MSK1. **H–J**. ELISA showed the effect of mutation of Thr-581 on LPS-induced TNFα, IL-6, and IL1-β production in activated astrocytes.

To further confirm whether MSK1 plays a role in LPS-induced inflammatory cytokine expression, total MSK1 expression was first diminished in astrocytes by transfecting with siRNA targeting MSK1. As predicted, siRNA also knocked down the protein expression of p-MSK1 (Thr581), induced by LPS, to 35% compared with control astrocytes or non-specific siRNA. In addition, the expression of iNOS induced by LPS was also inhibited by siRNA MSK1 (Fig. 6A, B). Next, the involvement of total MSK1 in the induction of cytokine expression by LPS was analyzed. Induction of TNFα, IL-6, and IL1-β expression by LPS was significantly increased in cells transfected with siRNA targeting MSK1, whereas no significant difference was seen in wild-type cells or cells transfected with a control siRNA (Fig. 6C–E). It has been reported that phosphorylation of Thr-581 in MSK1 is essential for the activation of the total MSK1 [25]. In this paper, we also demonstrated that p-MSK1 Thr-581 was significantly induced by LPS and tightly associated with the astrocyte inflammatory response. To determine whether p-MSK1 Thr-581 is a regulator of the astrocyte inflammatory response induced by LPS, we used plasmid expressing a mutant form of MSK1, in which the MSK1 phosphorylation site, Thr581, was inactivated by substituting with Ala (the T581A mutant). Mutation of Thr-581 to an alanine residue resulted in obviously decreased expression of p-MSK1 Thr-581 (Fig. 6F, G). As shown in Figure 6H–J, the involvement of p-MSK1 Thr-581 in the induction of various inflammatory cytokines in astrocytes by LPS was then analyzed. Induction of TNFα (Fig. 6H), IL-6 (Fig. 6I), and IL1-β (Fig. 6J) expression by LPS was significantly upregulated in cells transfected with Thr-581A, compared with wild-type cells.

## Discussion

Over the last decade, the concept of the brain as an immune-privileged organ has been rejected in light of unequivocal evidence that the permeability of the blood-brain barrier can be regulated under normal conditions and may become dysregulated in many chronic or acute CNS inflammatory processes [31], [32]. Overproduction by reactive astrocytes of proinflammatory cytokines, such as TNFα, IL-6, and IL1-β, can lead to delayed neuronal damage [33], [34]. Mitogen-activated protein kinases (MAPKs) play a key role in the regulation of cell proliferation, differentiation, survival, apoptosis, and the inflammatory response, as well as proinflammatory cytokine production [17], [18]. MSK1 is activated in vivo downstream of p38 and ERK. Although the role of total MSK1 in inflammatory gene expression has been studied extensively [23]–[26], it is now becoming apparent that MSK1 plays differing roles in different cells and different models. Indeed, some paradoxical conclusions have been reported recently. Some researchers found that MSK1 acted as an important negative regulator of inflammation following TLR activation [23], [35] while others suggested that a p-ERK1/2-MSK1- and p-NF-κB p65-dependent pathway was required for the highest induction of IL-6 in activated microglia [36]. Because MSK1 targets both pro- and anti-inflammatory genes, we cannot simply conclude that MSK1 functions in promoting inflammation or anti-inflammation genes. Additionally, because MSK1 activity is controlled by multiple phosphorylation sites, it seems necessary to explore the functions of the individual phosphorylation site in the inflammatory process.

In the present study, we studied the contribution of MSK1 and p-MSK1 Thr-581 in vivo and in vitro in LPS-induced inflammation in the CNS and examined the possible molecular mechanisms involved in MSK1 activation in LPS-treated astrocytes. Western blot analysis showed phosphorylated MSK1 (p-MSK1 Thr-581) was significantly induced after intracerebral injection of LPS into the lateral ventricles of the rat brain. Specific upregulation of p-MSK1 (Thr581) in astrocytes was also observed in the inflamed cerebral cortex. Additionally, in vitro studies indicated that upregulation of p-MSK1 may be involved in the subsequent astrocyte inflammatory process, following LPS challenge. Furthermore, in cultured primary astrocytes, both knock-down of total MSK1 by siRNA and specific mutation of Thr-581 of the MSK1 phosphorylation site resulted in much higher production of certain cytokines, such as TNFα and IL-6. Here, we reported for the first time that the p-MSK1 was involved in astrocyte inflammation, possibly inhibiting the production of inflammatory cytokines.

It was originally believed that only microglia, as the primary immune cells in the central nervous system (CNS), played a key role in inflammatory processes in the brain [37]. However, more and more evidence suggests that astrocytes, the major glial cells in the brain, are linked to many inflammatory responses of the CNS, and the responses of astrocytes to proinflammatory triggers might also be relevant, because of the impact that astrocytic responses might have locally on adjacent blood vessels [9], [13], [15], [38]. Under these circumstances, how to control the excessive astrocytic inflammatory response effectively could influence the outcome of the CNS inflammatory process.

Our results identified a new regulatory pathway for inflammatory cytokines, such as TNFα, IL-6, and IL1-β, during a LPS-induced astrocyte inflammatory response, based on the sequential activation of MAPKs and p-MSK1 (Thr-581). This pathway is a protein phosphorylation cascade with a wide range of cellular responses. In this paper, inhibition of p38 or ERK activity using pharmacological inhibitors blocked LPS-mediated phosphorylation of MSK1 and also blocked subsequent inflammatory cytokine release in astrocytes. That both SB203580 and PD98059 can inhibit cytokine production in astrocytes suggests that inhibitors of MAPKs block the phosphorylation of NF-κB, CREB, and chromatin H3, which are important transcription factors in the regulation of inflammatory genes downstream of MSK1 [21], [22], [39]. It has recently been established that MSK1 plays a role in inflammation and immunity. Also, phosphorylated MSK1 Thr581 is essential for MSK1 activity [25]. In macrophages, MSKs can regulate IL-10 production in response to LPS and thus influence TNFα/IL-6/IL-1β secretion. However, it was found that feedback mechanism is absent in fibroblasts [48]. Therefore, we will perform further experimentally test to explore whether this mechanism can still operate in astrocytes. H89, used as an inhibitor of MSK1, also inhibited the release of cytokines induced by LPS. However, a deficiency in MSK1, by either specific siRNA or mutation of p-MSk1Thr-581, resulted in higher production of TNFα, IL-6, and IL1-β. These findings were in contrast to the effect of H89 and the inhibitors of MAPKs. It may be explained that the reduced production of cytokines with H89 may result from its inhibition of PKA, in addition to MSK1 [40]. Besides, in addition to MSK1, various proteins have been identified as substrates of p38 and ERK1/2 pathway following inflammatory signaling. It has been proved that p38 regulate the LPS-induced activation of MK2 which is associated with the translational control of TNFα-mRNA in macrophages [46]. It may also be the explanation of the difference between the effect of MSK knockdown and inhibition of p38 and ERK in astrocytic inflammation. H89 has also been shown to target many other proteins other than kinases. And more importantly, H89 has multiple off-target effects [47]. This may explain why there was a partial inhibition of H89 on the activity of CREB in this study. This finding also suggests that MSK1 might be specifically involved in the activation of the negative feedback pathway, downstream of ERK and p38. However, the inherent mechanisms involved in the negative feedback regulation of MSK1 remain unclear. Some researchers have indicated that MSK1 may control the cellular localization of the upstream activators, ERK and p38, but not their protein expression [41]. It remains to be confirmed whether the same regulatory mechanisms apply during astrocyte inflammation in future studies.

MSK2 is closely related to MSK1 and, similar to MSK1, they contain the same kinase domains, including two distinct catalytic domains and relatively similar C- and N- terminal regions [25]. Besides, the regulation of MSK2 activation and that of MSK1 activation are similar [21], [23], [48], [49]. Therefore, in most cases, MSK1 and MSK2 play the similar roles. But, the expressions of MSK1 and MSK2 are not always the same in different cells. For example, MSK2 may not contribute to CREB phosphorylation in cortical neurons because it is expressed at low level [50]. However, in this paper, we mainly focus on the function of MSK1 in astrocytes after LPS treatment. With the development of research, we will know more detailed functions of MSK2 following injury.

Because the p38 pathway is critical for the immune and inflammatory responses, it has attracted considerable interest as a possible target for anti-inflammatory drugs [42], [43]. However, to date, none of these drugs has progressed through clinical trials because of unforeseen adverse events [44]. MSK1, activated by both p38 and ERK, is supposed to be a key inflammatory regulator [21], [22], [23], [24]. Also, in vivo experiments have demonstrated that mice lacking MSK1 are viable and do not develop any obvious health defect [21]. Thus, specific mutation of p-MSK1 phosphorylation sites may promote inflammation in the CNS. Regulation of MSK1 phosphorylation in activated astrocytes may provide a new strategy to protect against inflammation-induced astrogliosis.
